# Virtual reality simulation as a training tool for perfusionists in extracorporeal circulation: Establishing face and content validity

**DOI:** 10.1016/j.xjtc.2023.06.004

**Published:** 2023-06-20

**Authors:** Zaheer U.D. Babar, Samuel A. Max, Bryan G. Martina, Rodney A. Rosalia, Jette J. Peek, Antony van Dijk, Amir H. Sadeghi, Edris A.F. Mahtab

**Affiliations:** a Department of Cardiothoracic Surgery, University Medical Center, Erasmus MC, Rotterdam, The Netherlands; b Department of Cardiothoracic Surgery, Medisch Spectrum Twente, Enschede, The Netherlands

**Keywords:** virtual reality, extracorporeal circulation, simulator

## Abstract

**Objective:**

We conducted a prospective study to assess the face and content validity of a new virtual reality (VR) extracorporeal circulation simulator (ECC) developed for perfusionists to facilitate training and practice. We evaluated the opinions of students and staff members about the feasibility of the simulation. The 2 groups consisted of experts (qualified perfusionists) and novices (trainee perfusionists).

**Methods:**

Perfusionists (n = 12 experts and n = 11 trainees) received instructions on how to use the VR simulator and then proceeded to perform the start of cardiopulmonary bypass in the VR environment. Participants then completed a Usefulness, Satisfaction, and Ease of Use Questionnaire. The questions were rated on a 5-point Likert scale, ranging from 1 (fully disagree) to 5 (fully agree), to assess the face validity and content validity of this simulator.

**Results:**

Participants reported a predominantly positive experience with the VR-ECC simulator, with 96% (n = 22) agreeing that the simulator was a useful way of training ECC scenarios. All participants found it easy to interact with the software (100%, n = 23), and 82% of students (n = 9) believed it helped them remember the steps involved with initiating ECC. Finally, (87% [n = 20]) of participants believed the image quality and depth perception were good.

**Conclusions:**

Our next-generation simulator was valid for face and content constructs, and almost all participants found it to be a useful way of training for ECC scenarios. This simulator represents a first step toward truly blended digital learning and a new interactive, flexible, and innovative modality for perfusion training.

## Graphical abstract

**Figure:**
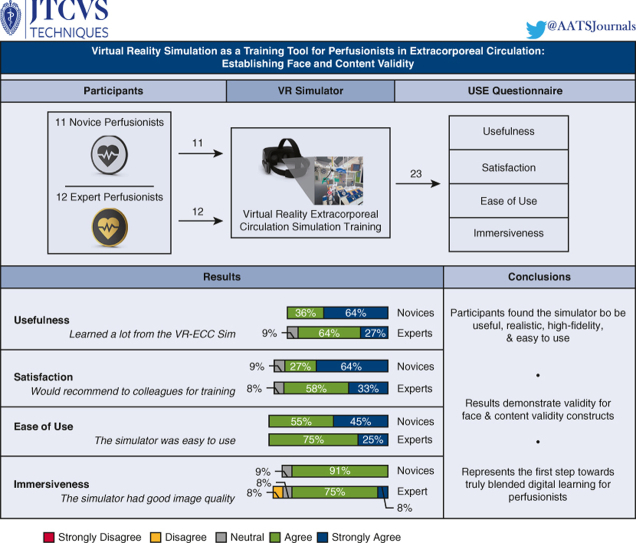

**Figure:**
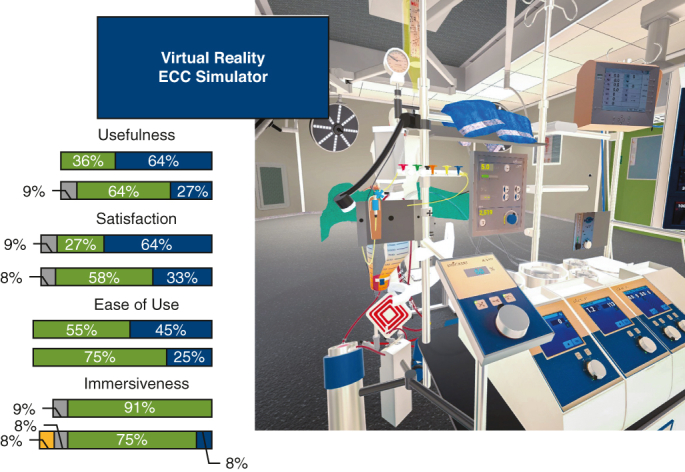
Virtual reality extracorporeal circulation simulator image and questionnaire results.

**Central Message**

Participants found the virtual reality extracorporeal circulation simulator to be useful to train on, realistic, easy to use, and contain high-fidelity visual models.

**Perspective**

Our realistic virtual reality extracorporeal circulation simulator affords new training opportunities for perfusionists of all levels. It will increasingly form part of future curricula to enable perfusionists to better prepare for the challenges of their profession. Our simulation will enable unlimited practice to master complex perfusion operations or prepare for unexpected disaster scenarios.

For trainee perfusionists, knowledge of physiology and the interaction between a patient's hemodynamic state and the heart-lung machine (HLM) is essential to prepare them for their clinical practice. A substantial part of a perfusionist's training process is dedicated to transferring this theoretical knowledge into practical skills. Providing this training presents logistical and financial challenges, not least during times of social distancing, travel restrictions, and health care budget constraints. The high cost of equipment and supplies puts considerable pressure on the training program budget, making it challenging to allocate enough resources for repetitive practice of complex procedures or emergency scenarios.

Virtual Reality (VR) simulation training may help to ameliorate these issues, and simultaneously provide equitable access to training across the globe. VR can provide fully immersive, interactive, and realistic scenarios in which the user can repeatedly train and acquire skills without the need to travel long distances or being present physically at a specific location. Other advantages of VR training for health care professionals include its convenience, flexibility, and cost-effectiveness.^1^ The efficacy of the VR modality in training (surgical) skills has previously been demonstrated.^2^, ^3^, ^4^

The application of a VR simulator in the education of perfusionists appears to be promising but remains to be fully investigated.^5^ The initial experience with augmented reality training of perfusion students demonstrated the significant value of this modality, although as of yet does not include a full HLM simulation.^1^ To the best of our knowledge, a fully virtual simulation has not yet been implemented in clinical perfusion training. VR can recreate fully immersive 360° interactive and realistic scenarios, enabling users to hone their skills without the need for expensive physical simulation equipment. Users can make mistakes without fear of consequences. Moreover, VR can be used in a multiuser setting, allowing different users to be present in the same scenario while physically distanced.^5^ Multiple studies have shown that simulation training effectively improves knowledge, confidence, motivation, and satisfaction with training versus standard training methods.^6^, ^7^, ^8^, ^9^ Creating an accurate virtual environment is essential to successfully transfer knowledge and skills, especially as an alternative conventional training method.^10^

To that end, we developed the VR extracorporeal circulation simulator (VR-ECC sim). In this simulator, we have recreated the process of preparing and building an HLM, the procedure for initiating cardiopulmonary bypass, the management of the patient while on bypass, and the procedure for weaning from bypass.

Once built, such a VR training tool requires validation to demonstrate its educational utility.^11^^,^^12^ This validation comprises different subtypes, including face validity and content validity.^13^ Face validity considers how realistic the simulator is, and how closely it resembles the look and feel of a physical HLM. This is judged by experts (qualified perfusionists) and nonexperts alike (novices/trainees).^11^ Content validity refers to the degree to which the content of a simulator precisely portrays the intended medical construct, in terms of knowledge or skills that it aims to impart through education.^13^ It is therefore assessed by examining the ratings that experts give in the usefulness and satisfaction sections of the questionnaire.

We performed a prospective study to assess the feasibility and to establish the face and content validity of the VR-ECC sim in a group of novice and expert perfusionists.

## Methods

### Study Design

#### Software development

We developed a vendor-agnostic VR simulation that recreates the perioperative steps, preparation, initiation, and termination of cardiopulmonary bypass using an HLM. Unreal Engine 4 (Epic Games) software was used for the simulation development, with visual modeling being performed in Autodesk Maya (Autodesk Inc).

**Figure 1:**
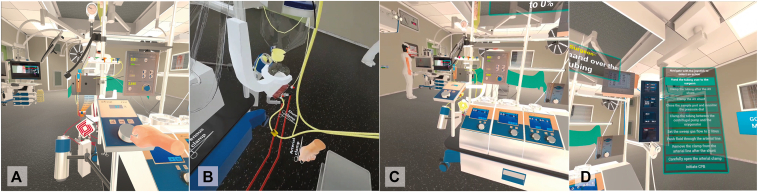
Screen captures from the virtual reality-extracorporeal circulation (VR-ECC) simulator, featuring from left-to-right: adjustment of the venous occluder (A), removal of the a clamp from the arterial line (B), an overview of the heart-lung machine (C), and the menu system by which users navigate through the simulation (D).

**Figure 2:**
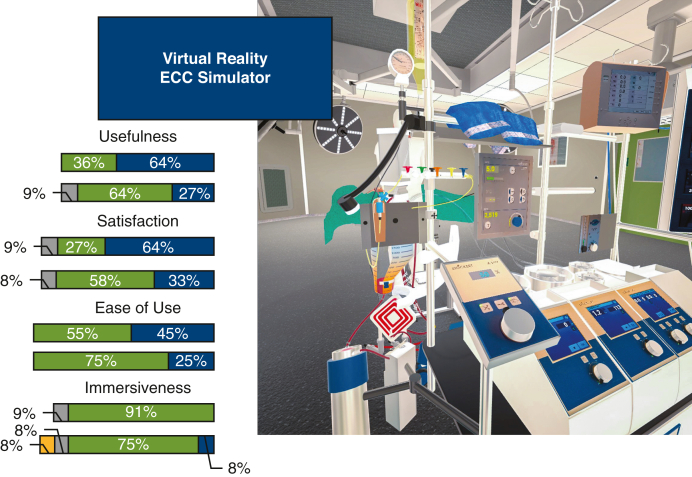
The central picture of this manuscript, displaying selected metrics from the use questionnaires, completed by experts and novices, and a screen capture from the virtual reality-extracorporeal circulation (*VR-ECC*) simulator.

The VR-ECC sim was designed by a multidisciplinary team consisting of perfusionists, VR software developers, digital transformation experts, and cardiothoracic surgeons from the cardiothoracic surgery departments at Erasmus Medical Centre, Distant Point LTD, and Medisch Spectrum Twente. Still images captured from the simulator can be found in Figures 1 and 2, and Video 1 shows the simulator.

The software used for this study was an alpha version of the simulator. One limitation of this early version was that users were not able to make mistakes. This feature will be included in future iterations of the simulator.

#### Hardware

A Meta Quest 2 (Meta Platforms Inc) head-mounted display, in combination with 2 VR controllers was used to run the VR-ECC sim in a standalone setup, without requiring the use of external computer hardware.

#### Interventions

**Figure 3:**
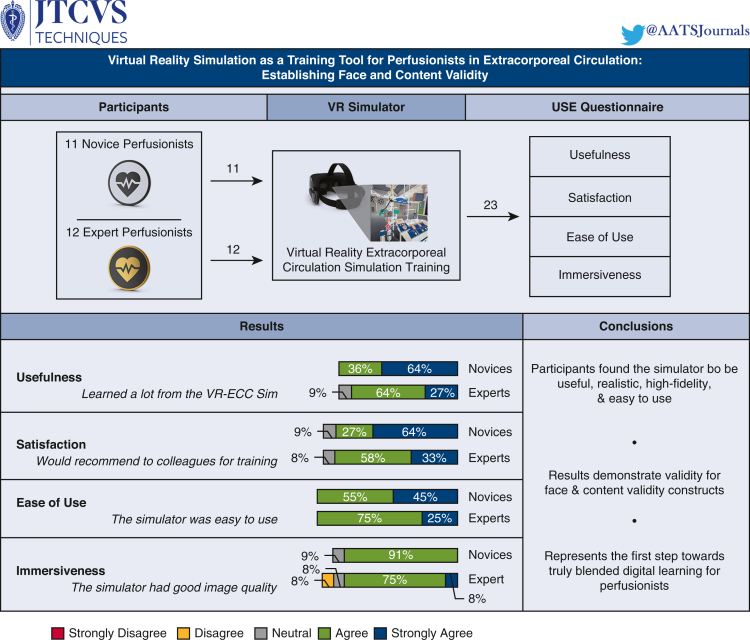
Graphical abstract showing a summary of the study design, as well as a sample of the results from each section of the Usefulness, Satisfaction, and Ease of Use Questionnaire. *VR*, Virtual reality; *VR-ECC*, virtual reality-extracorporeal circulation.

Participants were recruited through the Dutch national perfusion educational institute and through clinical perfusion departments in the Netherlands. The Institutional Review Board of the Erasmus MC University Medical Center approved the study protocol and publication of data with reference MEC-2023 to 0210 (April 28, 2023). As seen in Figure 3, a total of 11 novices (58% of the current national cohort of trainee perfusionists) and 12 experts were included and provided informed consent. A copy of the informed consent form can be found in Appendix E1. Before running the simulation, each participant was given a short briefing on the scenario, how to use the VR head-mounted display, and how to interact with the controls and software to perform the VR ECC sim. Participants then entered the simulation and were placed into the virtual operating theatre environment. They completed the “initiating cardiopulmonary bypass” scenario, which is interactive and contained a list of steps to be performed.

#### Data collection

Data was gathered through means of questionnaires given to participants that were completed after they completed the simulation. The validated Usefulness, Satisfaction, and Ease of Use Questionnaire format was employed to assess face validity and content validity specifically.^14^ This questionnaire employs four different themes to evaluate the reliability of new technologies and innovations on the subjects of Usefulness, Satisfaction, Ease of use, and Immersion. These questions were rated on a five-point Likert scale, ranging from 1 (fully disagree) to 5 (fully agree). In addition to the Usefulness, Satisfaction, and Ease of Use Questionnaire, demographic information and experience with ECC and digital learning platforms were gathered through a supplementary questionnaire. The end of the questionnaire contained three free text questions, where participants were asked to list the advantages and disadvantages of using VR-ECC sim as a training tool and to leave any comments they had about the experience of using the simulator. The questionnaire can be found in Appendix E2, and the raw data from the completed questionnaires can be found in the Online Data Supplement.

To examine face validity, we used questionnaire results from all participants on the ease of use and immersiveness. When assessing content validity, the results from the expert group were considered, specifically regarding usefulness and satisfaction.

### Statistical Analysis

Statistical analysis was performed using IBM SPSS Statistics for Windows, version 28.0 (IBM Corp). The χ^2^ test was used to perform statistical analyses of categorical data such as the participant characteristics. Continuous data are presented as mean ± SD, and categorical data, including Likert scales, are presented as percentages.

## Results

### Demographic Data

All participants (n = 23) who were enrolled in the study completed a questionnaire after completing the VR scenario. The baseline characteristics did not differ significantly between the two groups, with the exception of the group's exposure to digital learning or serious games, where the expert group had significantly more experience than the novice group. The breakdown of baseline characteristics and experience with virtual reality-related technologies can be found in Table 1.

**Table 1:** Baseline characteristics table showing the baseline characteristics of the participants in both groups, and statistical comparisons and their significance for each question

Baseline Characteristic	Novice perfusionists (n = 11)		Expert perfusionists (n = 12)		*P* value	Statistical test
Sex					0.37	χ^2^
Female	6	54.5	5	41.7		
Male	4	36.3	3	25.0		
N/A	1	9.0	4	33.3		
Age	29.5 ± 6.46		36.8 ± 11.5		.11	*t* test
Period as a trainee/expert (y)	1.35 ± 1.09		11.2 ± 13.0		.21	χ^2^
How many ECC operations have you participated in?					0.22	Fisher exact
1-5 times	2	18.2	0	0		
More than 10 times	9	81.8	12	100.0		
Do you have experience with gaming consoles (eg, computer gaming, Xbox, PlayStation)?					0.10	χ^2^
I have never used a gaming console	0	0	4	33.3		
I have used a gaming console a few times before	8	72.7	5	41.7		
I am gaming on a regular basis (at least once a month)	3	27.3	3	25.0		
How often do you use VR hardware/software (eg, VR gaming, simulations, consoles, entertainment etc.)?					0.16	Fisher exact
I have never had a VR experience until today	7	63.6	11	91.7		
I have used VR a few times before	4	36.4	1	8.3		
Do you have experience with physical simulation trainings in skills labs?					0.67	Fisher exact
I have never had simulation training before	4	36.4	3	25.0		
I have had simulation trainings multiple times before	7	63.6	9	75.0		
Do you have experience with digital training (eg, e-learning or serious games)?					0.02∗	χ^2^
I have never had such training before	7	63.6	2	16.7		
I have had a digital training a few times before	1	9.1	8	66.7		
I have had digital trainings multiple times before	3	27.3	2	16.7		
Do you have experience with a simulation training in VR?					N/A	
Yes	0	0.0	0	0		
No	11	100	12	100		

Values are presented as n (%) or mean ± SD. *N/A*, Not available; *ECC*, extracorporeal circulation; *VR*, virtual reality.

∗ Result is statistically significant where *P* < .05.

### Novices

**Figure 4:**
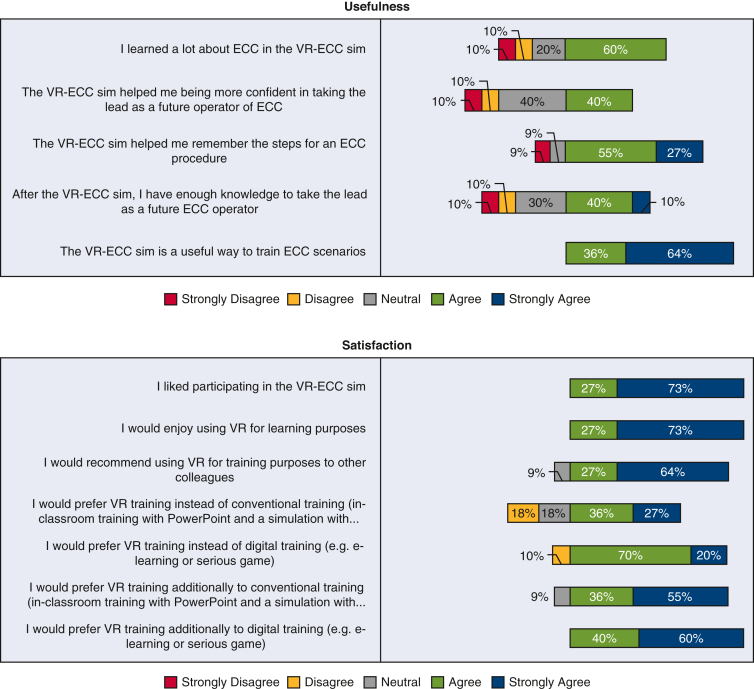

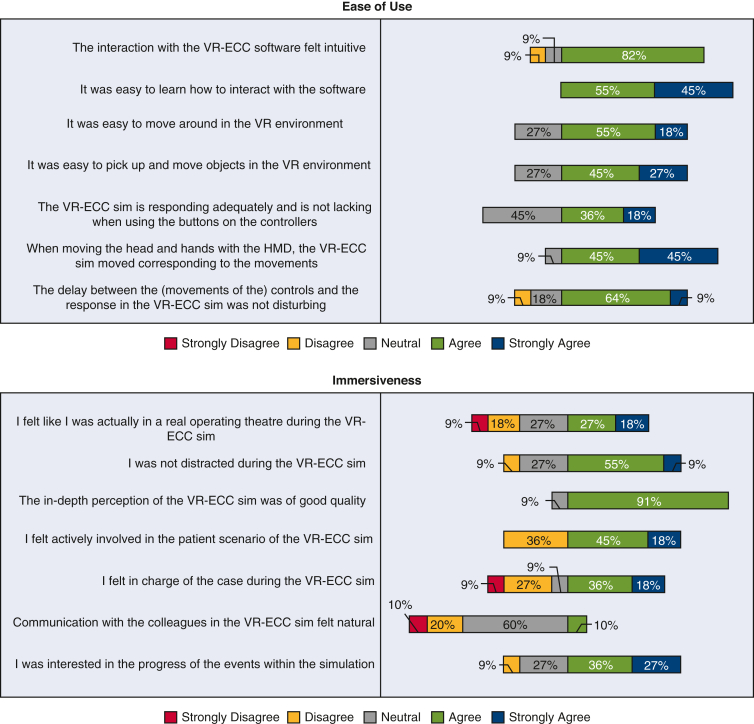
Stacked bar charts of the Usefulness, Satisfaction, Ease of Use Questionnaire data completed by novice perfusionists. *VR-ECC*, Virtual reality-extracorporeal circulation; *HMD*, head mounted displays.

Figure 4 shows the data collected from novice perfusionists after they completed the VR-ECC sim. Concerning the face validity, this group believed that the simulator was realistic, including 10 (91%) participants agreeing that the depth perception and image quality was good, 7 participants (63%) stating that they were not distracted from the simulation, and a further 7 participants (63%) believed they were actively involved in the ECC scenario. Most novice participants were interested in the progress of the simulation (n = 7 [63%]). Concerning content validity, novice participants believed the simulator was useful: the whole group (n = 11 [100%]) agreed that the VR-ECC sim was a useful training tool for perfusionists, 9 participants (82%) reported that the simulation helped them to remember the steps in performing an ECC, and 6 participants (60%) said that they learned a lot from performing the simulation. With respect to satisfaction, all novice participants (n = 11 [100%]) liked taking part in the VR-ECC sim and enjoyed using VR for learning purposes. Ten participants (91%) suggested that they would find the VR simulation useful in addition to conventional classroom training and 7 participants (63%) agreed that they would prefer VR simulation instead of conventional classroom training. Almost all novice participants (n = 10 [91%]) would recommend the VR-ECC sim to colleagues for training purposes. Regarding ease of use, all novice participants found it easy to interact with software (n = 11 [100%]), and 8 participants (73%) found it easy to move around the virtual environment. Most of the novice participants (n = 9 [90%]) agreed that their head and hand movements were sufficiently mirrored by the simulation. Finally, only 1 participant (10%) agreed that communication felt natural, which represents an area that should be addressed in future iterations of the simulator.

### Experts

**Figure 5:**
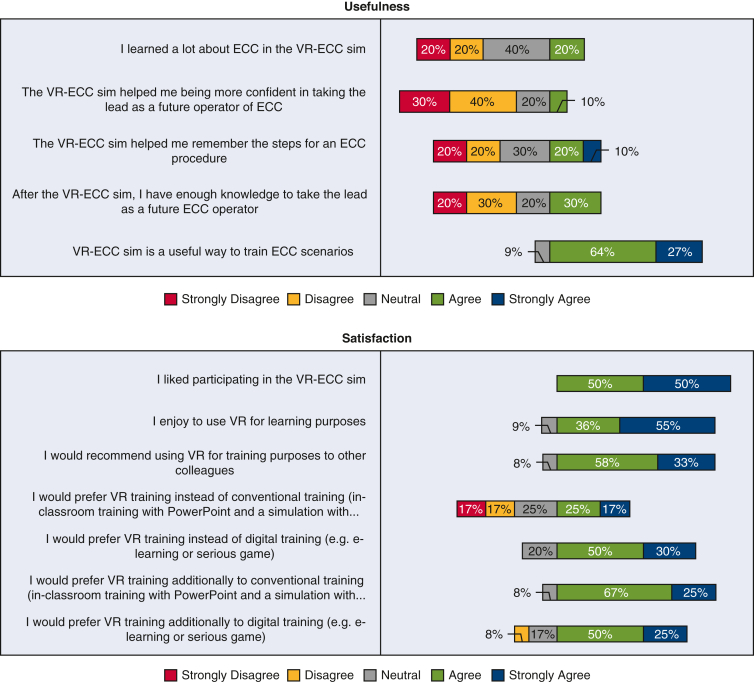

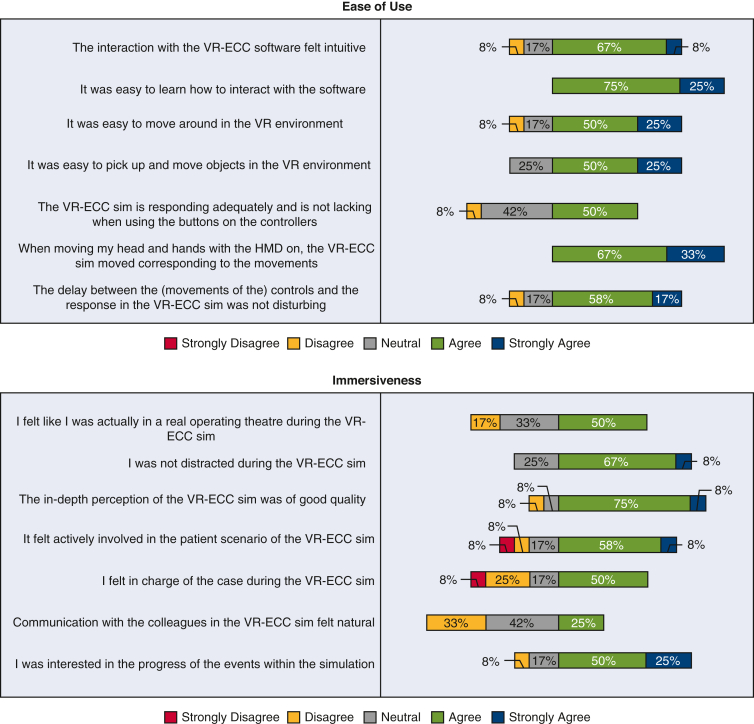
Stacked bar chart of the Usefulness, Satisfaction, Ease of Use Questionnaire data completed by expert perfusionists. *VR-ECC*, Virtual reality-extracorporeal circulation.

Figure 5 shows the data collected from expert perfusionists after they completed the simulation. The results obtained from the experts broadly mirrored those of the less experienced group. In terms of face validity, experts believed that the simulator was realistic, including 10 (83%) participants agreeing that the depth perception and image quality was good, 75% (n = 9) were not distracted from the simulation, and 66% (n = 8) believed they were actively involved in the patient scenario. The majority of expert participants were interested in the progress of the simulation (n = 9 [75%]). When considering content validity, the expert participants agreed with novice participants and believed the simulator was useful; almost the whole group (n = 10 [91%]) agreed that the VR-ECC sim was a useful training tool for perfusionists. Experts learned relatively little from the simulation, which is not unsurprising given their level of experience. Three participants (30%) reported that the simulation helped them to remember the steps in performing an ECC, and 2 participants (20%) said that they learned a lot from performing the simulation. Experts were satisfied with the VR-ECC sim, and all participants (n = 12 [100%]) liked taking part in the VR-ECC sim, and enjoyed using VR for learning purposes. Eleven participants (92%) suggested that they would find the VR simulation useful in addition to conventional Microsoft PowerPoint training and 5 participants (42%) agreed that they would prefer VR simulation instead of conventional PowerPoint training. Almost all expert participants (n = 11 [91%]) would recommend the VR-ECC sim to colleagues for training purposes. Regarding ease of use, all expert participants found it easy to interact with software (n = 11 [100%]), and 75% (n = 9) found it easy to move around the virtual environment. All experts (n = 12 [100%]) agreed that their head and hand movements were mirrored by the simulation. Only 25% (n = 3) agreed that communication felt natural.

All participants were asked to complete a free text section of the questionnaire addressing the advantages and disadvantages of the VR-ECC sim. Full results of this and the Usefulness, Satisfaction, Ease of Use Questionnaire data can be found in the Online Data Supplement. Common themes among the reported advantages included high image quality, a realistic simulation, the ability to practice procedure steps, establish a good routine, gain experience, practice with different systems, the ability to make mistakes without consequences, and to be able to troubleshoot difficult situations. Disadvantages included a (current) inflexibility in the order of steps, an unfamiliar HLM type in the simulation, and the slow progression of the simulation.

## Discussion

We present the results of this prospective feasibility study, which aimed to demonstrate the face and content validity of our prototype VR-ECC sim. The participants broadly agreed with all of the statements presented in the various categories of the USE Questionnaire (Usefulness, Satisfaction, Ease of Use, and Immersiveness). Therefore, this constitutes face validity, demonstrating that our simulator is realistic and that the simulated HLM looks and feels like a physical HLM.

Experts and novices alike felt immersed in the virtual environment, with only communication standing out as an area that should be improved in future iterations of the simulator. They also believed that the depth perception was good and were not distracted when performing the simulation. Almost all participants unanimously agreed that our VR-ECC simulation is a useful way to train for ECC scenarios and were very satisfied with the simulation experience. Experts and students alike found the simulator easy to use, with prior gaming/digital learning experience appearing not to be a factor in this regard. The study was conducted with an alpha version of the simulator. Future iterations of our simulator will contain more in-depth simulations allowing for mistakes with physiological consequences and troubleshooting scenarios with equipment failures and patients with challenging requirements. These scenarios will additionally be useful for surgeons to practice, especially for emergency situations requiring intense cooperation and communication between surgeons and perfusionists. We envision a multiuser simulator whereby a surgeon, scrub nurse, and perfusionist can practice in the same virtual operating theatre, creating a cohesive team before performing real cardiothoracic procedures.

When assessing content validity, we specifically considered the results from the expert group regarding the usefulness and satisfaction. The principal finding in this regard was whether or not experts believed that our simulator would be a useful way to train perfusionists, which they almost unanimously agreed that it was. Experts reported that they did not learn much from the simulation.

This outcome is not surprising, considering the high level of expertise of the expert group and the current aim of our simulator, which is to assist trainees in becoming familiar with the HLM and the operational environment of the operating theatre. In terms of satisfaction, experts rated the simulator highly, with a strong majority agreeing with every statement, with the exception of replacing conventional classroom/PowerPoint training with VR training, where 42% agreed. This may be in part due to the early stage of the simulator, and the current lack of theoretical knowledge imparted during such a training scenario. This is a feature that will be implemented in the future, such that a blended experience of simulation and theoretical learning can be produced, whilst leveraging the gold standard spaced repetition learning strategy.^15^

We envision that this training tool will form a part of blended learning curriculum in combination with traditional classroom learning, and hands-on clinical practice. Additionally, it will contribute to more radical educational transformation, including standardization, decentralization, and flexible learning strategies that can be performed at the convenience of the user. In this way, it can be used to increase and maintain competency. Evaluating user performance in the simulator will be key in this regard, such that users and supervisors alike can track progress and demonstrate competency in a proficiency-based curriculum.^16^

Creating such a curriculum will require a simulator that is flexible and can adapt to local practice in terms of equipment and standard operating procedure. Modeling different systems will require a visual component that is currently being produced, but also a physical model. Our aim is through use of real-life perfusion data, to build virtual models from a variety of equipment and clinical scenarios such that the simulator looks and responds like it would in a given clinical situation. With a realistic mechano-physiological HLM model, perfusionists can transition to new equipment and practice in a sandbox environment.

The multiplayer, multirole design of this simulator streamlines the learning of new center-specific protocols and the utilization of unfamiliar equipment, particularly in troubleshooting scenarios. It enables interaction between mentors and students within a simulated setting, allowing students to execute crucial tasks in a secure environment under expert supervision. Our VR simulation also helps strike a balance between theoretical knowledge and hands-on practical experience to ensure students are well prepared for real-life clinical situations.

The field of clinical perfusion is constantly evolving, with increasing complexity of clinical cases and ongoing advancements in technology. Although previous this study and previous studies have demonstrated the value of simulation in training perfusionists, the decision regarding which modality to choose—physical, augmented reality, or VR—is less clear. Equipment cost is likely to be a significant factor in this decision-making process. Current physical simulators, including the Califia Patient Simulator (Biomed Simulations) and ECCSIM (Senko Medical Instrument Mfg Co Ltd) cost in excess of 5 figures, with the latter cited as costing $40,000.^1^ Augmented reality is an order of magnitude less expensive, costing around $2100, but as of yet is not a complete simulation of an HLM.^1^

The start-up costs for the VR-ECC sim are estimated at $400 associated with the purchase of the VR headset. Our software will be provided as a software as a service, with setup, user licenses, and customer support covered via monthly subscriptions. Therefore, VR-ECC sim represents the most cost-effective option to our knowledge to provide spaced repetition learning of basic skills, as well as simulation training of novel techniques, enabling trainees to stay up to date with innovation in the field.

Software development costs often remain prohibitively high across the various platforms currently available, which represents an issue that should be addressed in future to ensure access to the benefits of next generation simulators is equitable. To that end, our subscription model and geographical pricing strategy greatly lowers entry barriers and accessibility to both individuals and organizations.

With this feasibility study, we demonstrate the VR-ECC sim fulfills the criteria for face and content validity. To validate the simulator, we will compare its educational performance to traditional training methods in terms of preparing perfusionists for disaster scenarios. This comparison will occur through a physical moulage scenario, and the outcomes measured will include time, accuracy, and safety, which will evaluate the simulator's construct validity. Once an intermediate difficulty version of the simulator is available, we will be able to assess participants' performance in the simulator with the aforementioned metrics. This will be rated by experts through a video made of the participant's test scenario in VR and thresholds for expert competency for each of these targets will be determined in advance. Finally, predictive validity, or the ability of the simulator to discriminate between novices and experts should be compared against the gold standard assessment's predictive ability. There is little consensus as to what is the gold standard of practical ECC assessment, making it difficult at this moment to produce such evidence.

### Limitations

Our limitations include a small group of participants because this was a feasibility study. We did not perform comparative statistics for the outcomes measured because there would not be sufficient data to do so, nor would this produce meaningful data. The simulator was still in the alpha stage of development and thus the versions tested by participants varied slightly with minor bug fixes implemented over the course of this study. Limitations of the simulator as reported by users included an inflexibility in the order of steps, an unfamiliar HLM type in the simulation, and slow progression through the simulation. These issues have largely been ameliorated in the new beta version of the simulator.

## Conclusions

To the best of our knowledge, this is the first VR simulator that recreates the setup and operation of an HLM for cardiopulmonary bypass. The results demonstrate that this simulator is valid in terms of face and content validity. Participants reported finding it useful, realistic, enjoyable, and easy to use. Future research should include a randomized controlled trial whereby the VR-ECC sim is compared with the gold standard classroom equivalent before performing a physical skills assessment where the time to completion and number of mistakes are assessed by an independent expert. With further development and testing, this high-fidelity simulator has the potential to become an essential part of the next generation of educational tools for perfusionists around the world.

### Conflict of Interest Statement

Drs Mahtab, Sadeghi, Babar, Max, Martina, Rosalia, and van Dijk and Ms Peek are co-developers of the VR-ECC sim.

The *Journal* policy requires editors and reviewers t disclose conflicts of interest and to decline handling or reviewing manuscripts for which they have a conflict of interest. The editors and reviewers of this article have no conflicts of interest.

## Figures and Tables

**Table:** 

		1. Fully disagree	2. Disagree	3. Neutral	4. Agree	5. Fully agree	N/A
Usefulness							
1	I learned a lot about ECC in the VR-ECC simulation						
2	The VR-ECC simulation helped me being more confident in taking the lead as a future operator of ECC						
3	The VR-ECC simulation helped me remember the steps in performing ECC						
4	After the VR-ECC simulation, I have enough knowledge to take the lead of a future ECC operation						
5	CPVR simulation is a useful way to train ECC scenarios						
Satisfaction							
6	I liked participating in the VR-ECC simulation						
7	I enjoy using VR for learning purposes						
8	I would recommend using VR for training purposes to other colleagues						
9	I would prefer VR training instead of conventional training (in-classroom training with PowerPoint and a simulation with multiple participants)						
10	I would prefer VR training instead of digital training (eg e-learning or serious game)						
11	I would prefer VR training additionally to conventional training (in-classroom training with PowerPoint and a simulation with multiple participants)?						
11	I would prefer VR training additionally to digital training (eg e-learning or serious game)						
Ease of use							
12	The interaction with the VR-ECC software felt intuitive						
13	It was easy to learn how to interact with the software						
14	It was easy to move around in the VR environment						
15	It was easy to pick up and move objects in the VR environment						
16	The VR-ECC simulation responds adequately and is not lacking when using the buttons on the controllers						
17	When moving the head and hands with the HMD, the VR-ECC simulation moved corresponding to the movements						
18	There delay between the (movements of the) controls and the response in the VR-ECC simulation was not disturbing						
Immersiveness							
19	I felt like I was actually in a real operating theatre during the VR-ECC simulation						
20	I was not distracted during the VR-ECC simulation						
21	The in-depth perception of the VR-ECC simulation was of good quality						
22	I felt actively involved in the patient scenario of the VR-ECC simulation						
23	I felt in charge of the case during the VR-ECC simulation						
24	Communication with the colleagues in the VR-ECC simulation felt natural						
25	I was interested in the progress of the events within the simulation						

## Appendix E2 User Experience Questionnaire for the Extracorporeal Circulation-Virtual Reality (ECC VR) Simulator

Please complete the following:

Male/female/other Age: __________ Profession: __________________________________

1. How many years of work experience do you have in thoracic surgery working as a perfusionist or trainee perfusionist? ____________________________________________________________
2. How many ECC operations have you participated in?
  • I have never participated in an ECC operation.
  • 1 to 5 times.
  • 5 to 10 times.
  • More than 10 times.
3. Do you have experience with gaming consoles (eg computer gaming, Xbox, PlayStation)?
  • I have never used a gaming console.
  • I have used a gaming console a few times before.
  • I game on a regular basis (at least once a month).
4. How often do you use VR hardware/software (eg VR gaming, simulations, consoles, entertainment etc.)?
  • I have never had a VR experience until today.
  • I have used VR a few times before.
  • I am experienced and use VR on a regular basis (at least once a month)
  • I am an VR expert (have a VR console and applications myself)
5. Do you have experience with physical simulation trainings in skills labs?
  • I have never had simulation training before.
  • I have had simulation trainings multiple times before.
  • I am a certified simulation trainer.
6. Do you have experience with digital training (eg e-learning or serious games)?
  • I have never had such training before.
  • I have had a digital training a few times before.
  • I have had digital trainings multiple times before.
7. Do you have experience with a simulation training in VR?
  • Yes.
  • No.

Write down the advantages and disadvantages of the VR-ECC simulation training, rank them in order of importance, from most important (1) to least important (3).

Advantages:

1.
2.
3.

Disadvantages:

1.
2.
3.

Do you have any comments or did you miss something in the simulation?

________________________________________________________________________

________________________________________________________________________

________________________________________________________________________

________________________________________________________________________

Thank you for participating!

## Supplementary Data

Online Data Supplement ECC Questionnaire Supplementary Revised

Video 1 A video captured from the virtual reality-extracorporeal circulation (VR-ECC) simulator showing a beta version of the simulator, where participants prepare the heart-lung machine and go on bypass. Video available at: https://www.jtcvs.org/article/S2666-2507(23)00198-0/fulltext.
