# Supplementary material for: Virtual reality simulation as a training tool for perfusionists in extracorporeal circulation: Establishing face and content validity
Source: JTCVS Tech. 2023 Jun 20;21:135–48. doi: 10.1016/j.xjtc.2023.06.004 (PMC10579814; doi:10.1016/j.xjtc.2023.06.004)
Supplement: Online Data Supplement — ECC Questionnaire Supplementary Revised [file mmc1.docx]

**User experience questionnaire for the VR-ECC simulator**

Please complete the following:

Male/female Age: __________ Profession: ________________________________

1. How many years of work experience do you have in thoracic surgery working as a perfusionist or trainee perfusionist? ________________________________________________________
2. How many ECC operations have you participated in?

- I have never participated in a ECC situation
- 1-5 times
- 5-10 times
- More than 10 times

1. Do you have experience with gaming consoles (e.g. computer gaming, Xbox, PlayStation)?

- I have never used a gaming console
- I have used a gaming console a few times before
- I game on a regular basis (at least once a month)

1. How often do you use VR hardware/software (e.g. VR gaming, simulations, consoles, entertainment etc.)?

- I have never had a VR experience until today
- I have used VR a few times before
- I am experienced and use VR on a regular basis (at least once a month)
- I am an VR expert (have a VR console and applications myself)

1. Do you have experience with physical simulation trainings in skills labs?

- I have never had simulation training before
- I have had simulation trainings multiple times before
- I am a certified simulation trainer

1. Do you have experience with digital training (e.g. e-learning or serious games)?

- I have never had such training before
- I have had a digital training a few times before
- I have had digital trainings multiple times before

1. Do you have experience with a simulation training in VR?

- Yes
- No

|  |  | 1. Fully disagree | 2. Disagree | 3. Neutral | 4. Agree | 5. Fully agree | N/A |
| --- | --- | --- | --- | --- | --- | --- | --- |
|  | **Usefulness** |  |  |  |  |  |  |
| 1 | I learned a lot about ECC in the VR-ECC simulation |  |  |  |  |  |  |
| 2 | The VR-ECC simulation helped me being more confident in taking the lead as a future operator of ECC |  |  |  |  |  |  |
| 3 | The VR-ECC simulation helped me remember the steps in performing ECC |  |  |  |  |  |  |
| 4 | After the VR-ECC simulation, I have enough knowledge to take the lead of a future ECC operation |  |  |  |  |  |  |
| 5 | The VR-ECC simulation is a useful way to train ECC scenarios |  |  |  |  |  |  |
|  | **Satisfaction** |  |  |  |  |  |  |
| 6 | I liked participating in the VR-ECC simulation |  |  |  |  |  |  |
| 7 | I enjoy using VR for learning purposes |  |  |  |  |  |  |
| 8 | I would recommend using VR for training purposes to other colleagues |  |  |  |  |  |  |
| 9 | I would prefer VR training instead of conventional training (in-classroom training with PowerPoint) |  |  |  |  |  |  |
| 10 | I would prefer VR training instead of digital training (e.g. e-learning or serious game) |  |  |  |  |  |  |
| 11 | I would prefer VR training additionally to conventional training (in-classroom training with PowerPoint)? |  |  |  |  |  |  |
| 11 | I would prefer VR training additionally to digital training (e.g. e-learning or serious game) |  |  |  |  |  |  |
|  | **Ease of use** |  |  |  |  |  |  |
| 12 | The interaction with the VR-ECC software felt intuitive |  |  |  |  |  |  |
| 13 | It was easy to learn how to interact with the software |  |  |  |  |  |  |
| 14 | It was easy to move around in the VR environment |  |  |  |  |  |  |
| 15 | It was easy to pick up and move objects in the VR environment |  |  |  |  |  |  |
|  |  | 1. Fully disagree | 2. Disagree | 3. Neutral | 4. Agree | 5. Fully agree | N/A |
|  | **Effectiveness** |  |  |  |  |  |  |
| 16 | The VR-ECC simulation responds adequately and is not lacking when using the buttons on the controllers |  |  |  |  |  |  |
| 17 | When moving the head and hands with the HMD, the VR-ECC simulation moved corresponding to the movements |  |  |  |  |  |  |
| 18 | There delay between the (movements of the) controls and the response in the VR-ECC simulation was not disturbing |  |  |  |  |  |  |
|  | **Immersiveness** |  |  |  |  |  |  |
| 19 | I felt like I was actually in a real operating theatre during the VR-ECC simulation |  |  |  |  |  |  |
| 20 | I was not distracted during the VR-ECC simulation |  |  |  |  |  |  |
| 21 | The in-depth perception of the VR-ECC simulation was of good quality |  |  |  |  |  |  |
| 22 | I felt actively involved in the patient scenario of the VR-ECC simulation |  |  |  |  |  |  |
| 23 | I felt in charge of the case during the VR-ECC simulation |  |  |  |  |  |  |
| 24 | Communication with the colleagues in the VR-ECC simulation felt natural |  |  |  |  |  |  |
| 25 | I was interested in the progress of the events within the simulation |  |  |  |  |  |  |

Write down the advantages and disadvantages of the VR-ECC simulation training, rank them in order of importance, from most important (1) to least important (3).

**Advantages:**

1.

2.

3.

**Disadvantages:**

1.

2.

3.

Do you have any comments or did you miss something in the simulation?

__________________________________________________________________

__________________________________________________________________

__________________________________________________________________

__________________________________________________________________

Thank you for participating!

**Results on the questionnaires of the Face validity and content validity of all participants**

**Table 1: Face Validity**

|  | **VR ECC Scenario** | | | | **Total** | |
| --- | --- | --- | --- | --- | --- | --- |
|  | Experts | | Novices | |  |  |
|  | (n=12) | | (n=11) | | (n=23) | |
|  | Median | IQR | Median | IQR | Median | IQR |
| **Ease of use** |  |  |  |  |  |  |
| Q13: The interaction with the VR-ECC software felt intuitive | 4 | 0.75 | 4 | 0 | 4 | 0 |
| Q14: It was easy to learn how to interact with the software | 4 | 0.75 | 4 | 1 | 4 | 1 |
| Q15: It was easy to move around in the VR environment | 4 | 1.5 | 4 | 1 | 4 | 1 |
| Q16: It was easy to pick up and move objects in the VR environment | 4 | 1.5 | 4 | 2 | 4 | 2 |
| **Effectiveness** |  |  |  |  |  |  |
| Q17: The VR-ECC simulation responds adequately and is not lacking when using the buttons on the controllers | 3.5 | 1 | 4 | 1 | 4 | 1 |
| Q18: When moving the head and hands with the HMD, the VR-ECC simulation moved corresponding to the movements | 4 | 1 | 4 | 1 | 4 | 1 |
| Q19: There delay between the (movements of the) controls and the response in the VR-ECC simulation was not disturbing | 4 | 0.75 | 4 | 1 | 4 | 1 |
| **Immersiveness** |  |  |  |  |  |  |
| Q20: I felt like I was actually in a real operating theatre during the VR-ECC simulation | 3.5 | 1 | 3 | 2 | 3 | 1 |
| Q21: I was not distracted during the VR-ECC simulation | 4 | 0.75 | 4 | 1 | 4 | 1 |
| Q22: The in-depth perception of the VR-ECC simulation was of good quality | 4 | 0 | 4 | 0 | 4 | 0 |
| Q23: I felt actively involved in the patient scenario of the VR-ECC simulation | 4 | 1 | 4 | 2 | 4 | 2 |
| Q24: I felt in charge of the case during the VR-ECC simulation | 3.5 | 2 | 4 | 2 | 4 | 2 |
| Q25: Communication with the colleagues in the VR-ECC simulation felt natural | 3 | 1.75 | 3 | 1 | 3 | 1 |
| Q26: I was interested in the progress of the events within the simulation | 4 | 1.5 | 4 | 2 | 4 | 2 |

| **Table 2: Content Validity Results** | |  |  |  |  |  |  |
| --- | --- | --- | --- | --- | --- | --- | --- |
|  | Experts | | Novices | | Total | |  |
|  | (n = 12) | | (n=11) | | (n=23) | |  |
|  | Median | IQR | Median | IQR | Median | IQR | P value* |
| **Usefulness** |  |  |  |  |  |  |  |
| Q1: I learned a lot about ECC in the VR-ECC simulation | 3 | 1.5 | 4 | 1.25 | 3 | 2 | 0.143 |
| Q2: The VR-ECC simulation helped me being more confident in taking the lead as a future operator of ECC | 2 | 2 | 3 | 1.25 | 3 | 1.7 | 0.043 |
| Q3: The VR-ECC simulation helped me remember the steps in performing ECC | 3 | 2.25 | 4 | 1 | 4 | 1.5 | 0.51 |
| Q4: After the VR-ECC simulation, I have enough knowledge to take the lead of a future ECC operation | 2.5 | 2.25 | 3.5 | 1.25 | 3 | 2 | 0.218 |
| Q5: The VR-ECC simulation is a useful way to train ECC scenarios | 4 | 1 | 5 | 1 | 4 | 1 | 0.116 |
| **Satisfaction** |  |  |  |  |  |  |  |
| Q6: I liked participating in the VR-ECC simulation | 4.5 | 1 | 5 | 1 | 5 | 1 | 0.379 |
| Q7: I enjoy using VR for learning purposes | 5 | 1 | 5 | 1 | 5 | 1 | 0.438 |
| Q8: I would recommend using VR for training purposes to other colleagues | 4 | 1 | 5 | 1 | 4 | 1 | 0.288 |
| Q9: I would prefer VR training instead of conventional training (in-classroom training with PowerPoint and a simulation with multiple participants) | 3 | 2 | 4 | 2 | 4 | 2 | 0.228 |
| Q10: I would prefer VR training instead of digital training (e.g. e-learning or serious game) | 4 | 1.25 | 4 | 0.25 | 4 | 0.75 | 0.912 |
| Q11: I would prefer VR training additionally to conventional training (in-classroom training Q13: with PowerPoint and a simulation with multiple participants)? | 4 | 0.75 | 5 | 1 | 4 | 1 | 0.288 |
| Q12: I would prefer VR training additionally to digital training (e.g. e-learning or serious game) | 4 | 1.5 | 5 | 1 | 4 | 1 | 0.080 |
| * Mann-Whitney U test |  |  |  |  |  |  |  |

**Supplementary file**

Advantages and disadvantages of the VR-ECC sim from the questionnaire as reported by the participants

**Participant 1 (Novice)**

Advantages:

1. You can practice the most complex situations in a safe environment
2. It’s an easy hand-on training to learn the steps/basics of ECC
3. More helpful way to remember steps by doing it, instead of reading

Disadvantages:

1. You can’t make a mistake
2. Some steps went to fast
3. You cannot train communication, only steps.

Comments:

I haven’t learned a lot. But I did fill in “agree”. This is because I do see the potential of CVR in learning and training simulation.

**Participant 2 (Novice)**

Advantages:

1. Learn the procedure
2. Feel more confident

Disadvantages:

1. “Slangen onsteriel aangeven” – tubing handed over without preserving sterility
2. “Soms te lang wachten op volgende stap” – Sometimes had to wait for too long for the next step

Comments:

**Participant 3 (Novice)**

Advantages:

1. Fault scenario training
2. No real-life consequence when something goes wrong.
3. Repetition possible

Disadvantages:

1. Hard to program all different scenarios
2. Expensive equipment
3. Every center has different material and protocols. Hard to make it general.

Comments:

- Connection between devices, measurements, patient
- Communication: what is a question or remark?

**Participant 4 (Expert)**

Advantages:

1. Training on not living object
2. You could build some issues with perfusion in it so you can learn from it
3. You can train anytime or anywhere

Disadvantages:

1. It costs some time to develop to a great level
2. You need some space to make use of it (safely)
3. It’s more inactive communication rather then active communication

Comments:

**Participant 5 (Novice)**

Advantages:

1. Train Cases
2. Get feeling with performing ECC

Disadvantages:

1. The list popped up so fast, that I did not have to think by myself what the next steps would be
2. When you do an incorrect task, Venous occluder or RPM I was forgotten, the VR simulation moved forward. So it did not see the mistakes

Comments:

- Walking through the space around the HLM (as a box) so that can remove the clamps by yourself instead of by choosing the tasks

**Participant 6 (Novice)**

Advantages:

1. “Prima tool om beginnende perfusionisten mee te trainen” – Great tool to train novice perfusionists with.

Disadvantages:

1. “Tekst langer in beeld: genoeg tijd om je taak te leren” – Keep the text on the screen for longer: enough time to learn your task.

Comments:

**Participant 7 (Novice)**

Advantages:

1. “Je leert de goede volgorde van handelingen” – You learn the correct order of steps
2. “Het is net echt dus beter om te oefenen voordat je echt op bypass gaat” - It's very realistic so it’s better to practice before you really go on bypass
3. “Heel leerzaam” – very educational

Disadvantages:

1. “Niet gewend aan deze configuratie qua pack en machine” – Not used to this configuration in terms of the pack and machine

Comments:

**Participant 8 (Novice)**

Advantages:

1. “Goede aanvulling op lessen” – Good supplement to lessons
2. “Zou goed zijn om ingrepen te oefeenen die niet vaak voorkomen” – Would be good to practice interventions that are infrequently performed
3. “Goed beeld, hebt wel gevoel dat je in OK zit” – Good image; you get the feeling that you really are in the operating theatre

Disadvantages:

1. “Verschilt nogal per hartcentrum hoe/waarmee en welke stappen gewerkt wordt” – It differs quite a bit per heart centre how / with what and which steps are worked

Comments:

**Participant 9 (Novice)**

Advantages:

1. Easy accessibility
2. Fast training of difficult and rare cases

Disadvantages:

1. Still in start up phase

Comments:

- Lots of potential

**Participant 10 (Novice)**

Advantages:

1. “Het oefenen van het starten van bypass” – Practicing initiating bypass
2. “Stappen leren zonder enige consequenties” - Learning steps without any consequences
3. “Training van calamiteiten (toekomst)” – Training for emergencies (future)

Disadvantages:

1. “Tekst was niet helemaal duidelijk te lezen” – On-screen text was not completely clear
2. “Op dit moment alleen witoefen van de taken; geen eigen keuzes maken” – Currently only dry run of the tasks; you can’t make your own choices
3. “Soms ondelijk welke knop gebruikt moet worden voor openen klem etc.” – Occassional uncertainty regarding which button to use to open clamp etc.

Comments:

**Participant 11 (Novice)**

Advantages:

1. “Veilige manier om te oefenen/kennis te maken met het starten van ECC” - Safe way to practice/know how to start ECC
2. “Goede bewust wording van de benodigde stappen”- Good way to become aware of the necessary steps.
3. “Bekend raken met waar alle knoppen zitten en wat ze doen" – Can familiarise yourself with where all the controls are and what they do.

Disadvantages:

1. “Je kunt het nu niet fout doen (nog niet)” - You can’t make any mistakes (yet)
2. “De communicatie / rol in het team is wat passief” – The commucation and role of the user in the team is a bit passive
3. “Misschien dat niet iedere gebruiker even handig is met technologie waardoor zij minder aan de training hebben” - It’s possible that not every user is equally adept with technology, such that they benefit less from this training

Comments:

“Het zal tof zijn als er in de toekomst consequenties zitten aan de handelingen (flow en drukverandering) en meer volume schommelingen.” – It would be great if in the future there are consequences to the actions (flow and pressure changes) and more volume fluctuations.

**Participant 12 (Expert)**

Advantages:

1. “Goede volgorde van events” – Good sequence of events

Disadvantages:

1. “level + art klem?” – Level + arterial clamp?
2. “flow fluctueert heel snel tussen 0 en 3000rpm” – flow fluctuates very quickly between 0 and 3000 rpm
3. “timer ECC werkt niet.” – timer for the ECC isn’t working

Comments:

- “Zuigers benamingen niet juist” – Suction pumps are named incorrectly
- “Flow bij druk & pulsaties”- Flow with pressure and pulsations
- “Klemmen op haken?” Clamps on hooks?

**Participant 13 (Expert)**

Advantages:

1. Good preparation for the students before work in the OR.
2. Focus on the progress of action in OR
3. Reproducibility

Disadvantages:

1. Lack of flexibility
2. Simplicity of actions - so far

Comments:

**Participant 14 (Expert)**

Advantages:

1. “Spoed situaties trainen” – practice emergency situations
2. “Volgorde oefenen” – practice order of steps

Disadvantages:

1. “Op OK kunnen dingen anders gaan” – in theatre, things may go differently

Comments:

- “Sommige dingen moeten op een specifieke volgerde” – Some things must go in a specific order
- “Niet altijd duidelijk welke stappen precies moeten” – Not always clear which steps are required

**Participant 15: (Expert)**

Advantages:

1. Program responded good on your actions.
2. It could be useful for people with no ECC experience
3. Video quality is very good.

Disadvantages:

1. No free choice in what your next step will be
2. You cannot walk around the HLM
3. You cannot make mistakes

Comments:


**Participant 16 (Expert)**

Advantages:

1. Build a nice routine without pressures from others
2. Easy introduction to OR setting
3. Innovative way of learning

Disadvantages:

1. You can get nauseous
2. Expensive to develop and make adjustments
3. You have to create a safe environment for everyone + assistance if necessary

Comments:

**Participant 17 (Expert)**

Advantages:

1. Very realistic and in 3D training a lot of details
2. This VR has a lot of future potential with several case scenarios
3. You are able to let everyone feel the world of perfusion

Disadvantages:

1. There is no possibility to make a mistake
2. There is only one order to do the steps

Comments:

I think it’s a superb start and there is a lot of potential.

**Participant 18 (Expert)**

Advantages:

1. “Je kunt vlieguren maken.” – you can build up your flight hours (experience)
2. “Je kunt moelijke situaties “droog” trainen” – you can have a dry run for difficult situations
3. “Het is leuk om te doen” – it’s fun to do

Disadvantages:

1. “Je kunt nog niet zelf je handelingen bepalen” – You can’t yet decide on your own order of actions performed
2. “je kunt niet zlef bewegen in de VR setting” – you can’t move yourself in the VR setting

Comments:

- “Gebuik middelvinger versus wijsvinger om iets vast te pakken is onhandig” – Using your middle finger versus you index finger to grab something is awkward.
- “Er zijn 3 rechter zuigers te zien” – There are 3 suckers visible on the right
- “In de rechter zuiger ligt geen tubing” – There is no tubing in the right sucker
- “Soms is niet duidelijk dat je rechts op instructies moet kijken; Soms is dat namelijk niet.” – Sometimes it’s not clear that you should look to the right for the instructions – sometimes that’s not the case.

**Participant 19 (Expert)**

Advantages:

1. Not harmful for patient when mistakes are made.
2. Easy to practice difficulties
3. Can use as much as you want

Disadvantages:

1. Not yet possible to practice with surgeon at the same time
2. There is no feedback with your touches on the tubes
3. You need to wait on the program from one step to the next

Comments:

**Participant 20 (Expert)**

Advantages:

1. “Realistisch oefenen in VR omgeving” – Practice in realistic VR environment
2. “Oefenen met andere systemen” – Practice with different (HLM) systems
3. “Leren troubleshooten met gevaarlijke situaties.” – Learn to troubleshoot in dangerous situations.

Disadvantages:

1. “Wat traag, tijdens ECC komen events sneller na elkaar.” – A bit slow, as during ECC events follow each other more quickly.
2. “Acties perfusionist komen nadat perfusionist het vermeldt” – Perfusionists actions occur after the perfusionist reports having done them
3. “Ben meer commandos gewend in plaats van volzinnen.” – Am used to shorter commands instead of full sentences.

Comments:

**Participant 21 (Expert)**

Advantages:

1. “Prachtige tool voor opleiding” – Wonderful tool for training
2. “Heel realistisch” – Very realistic
3. “Stappenplan geeft duidelijk overzicht en structuur” – Step-by-step plan provides a clear overview and structure.

Disadvantages:

1. “Mist interactie met andere disciplines” – Misses interaction with other disciplines
2. “Soms wat traag” – Occasionally a bit slow
3. “Namen op de pompen moeten nog worden aangepast.” – Names on the pumps still need to be adjusted.

Comments:

**Participant 22 (Novice)**

Advantages:

1. Errors have no real consequences. There is room for trying things (if the software allows it).
2. There is room for taking your time in learning the steps.
3. Feels more real than a normal game-setting. But it’s no the real thing.

Disadvantages:

1. The pressure of a real setting is gone (which is also an advantage)
2. The simulations gives the illusion that each case will work like that. No room for improvisation in case something deviates from ‘normal’!
3. There is just one setup of the system. Not everyone has that setting/setup.

Comments:

- I would like to sit on the chair/stool instead of on the floor in the simulation
- The simulation is rigid. There is for example, only 1 right place to place a clamp.
- The order of things is not exactly right

**Participant 23 (Expert)**

Advantages:

1. Learning routine steps
2. Learning to analyze before taking action
3. Learning the layout of the specific HLM!

Disadvantages:

1. Precise placement of clamps not clear (area)
2. WiFi depedent for continuation
3. After completing an action, it was uncertain if the action was successful.

Comments:

- Great experience! Loved it. I believe this to have great implications e.g. new colleagues can learn new setups and new HLMs! (Other brands)
