# Supplementary figures and images for: Virtual reality simulation as a training tool for perfusionists in extracorporeal circulation: Establishing face and content validity
Source: JTCVS Tech. 2023 Jun 20;21:135–48. doi: 10.1016/j.xjtc.2023.06.004 (PMC10579814; doi:10.1016/j.xjtc.2023.06.004)

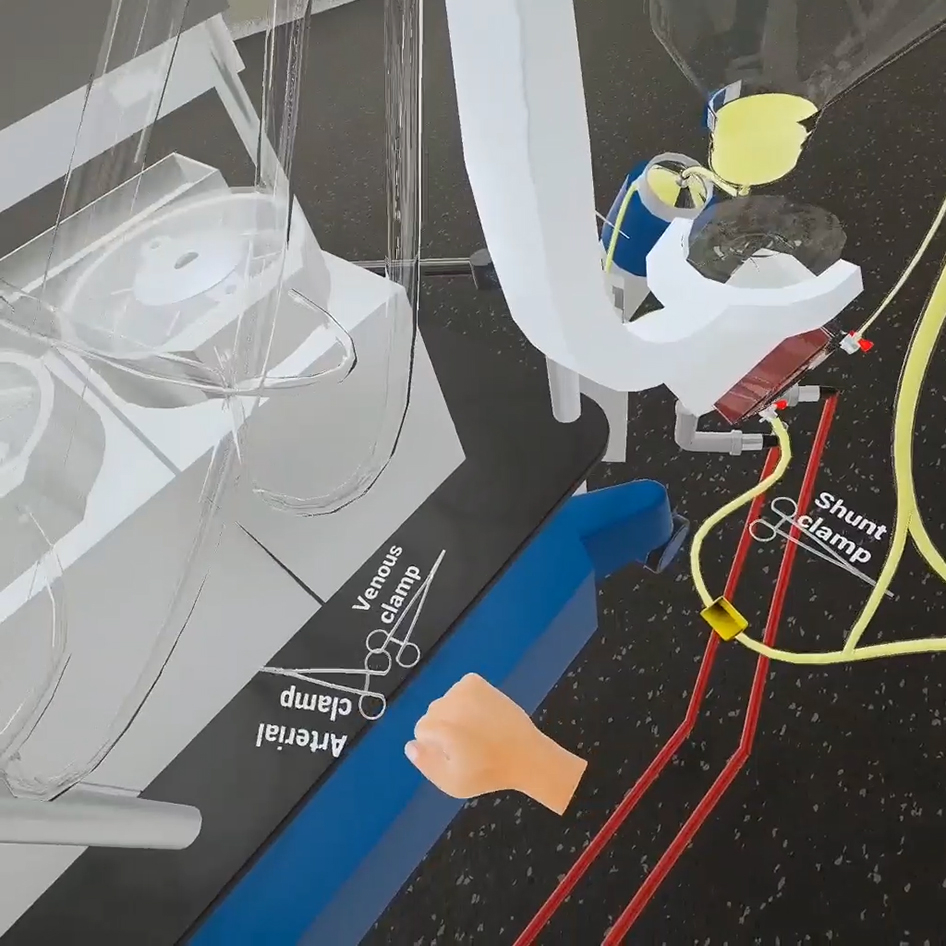

Supplement: Video 1 — A video captured from the virtual reality-extracorporeal circulation (VR-ECC) simulator showing a beta version of the simulator, where participants prepare the heart-lung machine and go on bypass. Video available at: https://www.jtcvs.org/article/S2666-2507(23)00198-0/fulltext. [file fx2.jpg]
